# An exploratory study of students’ perceptions of advice and support services in a science university

**DOI:** 10.3389/fmed.2025.1660132

**Published:** 2025-09-03

**Authors:** Abdulaziz Mohammed Althewini

**Affiliations:** ^1^College of Science and Health Professions, King Saud bin Abdulaziz University for Health Sciences, Riyadh, Saudi Arabia; ^2^Ministry of National Guard Health Affairs, King Abdullah International Medical Research Center (KAIMRC), Riyadh, Saudi Arabia

**Keywords:** college education, higher education, education quality, student perception, student satisfaction

## Abstract

This exploratory qualitative study investigates the experiences of health sciences students with academic advising in an English as a Medium of Instruction (EMI) context. Through virtual semi-structured interviews with four students, the study identifies preliminary themes: limited accessibility of support services, the need for personalized advising, cultural and language influences, and the role of technology in academic support. Students expressed a preference for discussing complex issues in their native Arabic language, highlighting the importance of linguistic and cultural sensitivity. While AI-based advising tools improve accessibility, participants emphasized the irreplaceable value of human-centered, empathetic guidance. Given the small sample size, the findings do not claim generalizability or thematic saturation but are intended to offer early, exploratory insights that may inform future research. The study recommends blended advising models that integrate technology with personalized support and calls for culturally responsive advisor training to enhance student satisfaction and success.

## Introduction

In the evolving landscape of higher education, particularly within the Global South, student satisfaction has become a key indicator of institutional quality and accountability. In Saudi Arabia, this focus is intensified by national transformation initiatives like *Vision 2030*, which place increasing pressure on universities to enhance not only educational outcomes but also the overall student experience. As the Kingdom seeks to align its higher education sector with global standards while preserving cultural identity, universities are expected to meet rising expectations related to educational quality, student-centered services, and academic advising.

One of the most powerful yet underleveraged mechanisms for supporting student success is academic advising. Beyond course selection, advising plays a vital role in fostering engagement, supporting retention, and guiding students through personal and academic challenges. Prior research has linked effective advising to stronger academic outcomes, a greater sense of belonging, and lower attrition rates—particularly among first-year students who are navigating significant transitional stress (1, 2). While academic advising is widely acknowledged as a valuable component of student success, advising systems in many Saudi universities face ongoing challenges related to administrative complexity, limited staffing, and varying degrees of cultural and linguistic alignment. These challenges are particularly pronounced in science-focused programs, where students often navigate demanding coursework, structured curricula, and significant career-related pressures.

### Advising in EMI contexts: a double burden

This problem is compounded in institutions where English is the official medium of instruction (EMI)—a growing trend across Saudi universities. The adoption of EMI reflects a strategic effort to increase academic competitiveness, attract international recognition, and prepare graduates for participation in the global workforce (3, 4). However, this shift has created a hidden layer of difficulty for students who must now navigate both academic and advisory interactions in a second language.

Although EMI is often viewed as a symbol of progress, its implementation in non-native English-speaking contexts introduces significant friction. Linguistic limitations among students can make it difficult to comprehend course content, communicate concerns clearly, or seek academic help—especially in one-on-one settings like advising. Likewise, many faculty members and advisors may lack the pedagogical and linguistic training to support students effectively in English, resulting in communication gaps and reduced emotional attunement (5, 6).

Critically, EMI creates an invisible disadvantage for students seeking personalized support. Expressing nuanced emotions, personal dilemmas, or academic confusion requires a level of comfort and fluency that many students do not possess. When academic advising is delivered exclusively in English, students may hesitate to engage fully, censor their concerns, or walk away with unresolved questions. This disconnect is not simply linguistic—it is emotional, cognitive, and cultural.

### The cultural mismatch

The EMI context is further complicated by the sociocultural context of Saudi Arabia, where Arabic is not only the native language but also tightly linked to cultural and familial norms. Research shows that students, parents, and faculty often experience tension between the institutional push for English proficiency and a collective desire to preserve cultural identity (6). These tensions surface most acutely in moments of vulnerability—such as when a student must disclose academic struggles to an advisor in English, or when a student cannot find the words to explain anxiety, confusion, or failure in a foreign language.

The consequences are clear: when language and culture are not accommodated in advising settings, students may feel alienated, disengaged, and unsupported. Ironically, the very systems intended to help students succeed may unintentionally exclude the most at-risk learners—those with lower English proficiency, those from satellite campuses, and those whose cultural expectations clash with institutional norms.

### Research gap and aim

While numerous studies have examined the structural and policy dimensions of EMI in Saudi higher education, few have investigated how students *experience* academic advising within this setting. Existing research often relies on quantitative data, offering broad trends but missing the complexity of lived experience. There is a critical need to understand how students navigate the dual challenge of seeking academic advice in a second language within a culturally layered environment.

This study addresses that gap by qualitatively exploring the perspectives of health sciences students in an EMI university in Saudi Arabia. Through in-depth interviews, it examines how language, culture, advisor behavior, and institutional structure intersect to shape the advising experience. Rather than claiming generalizability, this exploratory case study aims to generate early insights that can inform the design of more culturally and linguistically inclusive support systems in Saudi higher education.

## Methods

### Research design

This study adopts a qualitative research design to explore the deep, subjective experiences of health sciences students. A phenomenological approach is employed to understand how participants interpret and make sense of their interactions with academic advice and support services. Semi-structured virtual interviews are used as the primary data collection method, allowing for flexibility in exploring participants’ perspectives while maintaining a focus on key research questions.

### Participant selection

The study involves a purposive sample of four health sciences students, deliberately chosen to create a small, manageable group that allows for an in-depth exploration of their individual experiences with academic advice and support (Table 1). Participants are selected based on specific criteria to ensure diversity in gender, aiming for a balanced representation of male and female students, as well as variation in campus location to capture potential differences in institutional resources or culture. Additionally, participants are drawn from different academic group affiliations within the health sciences, including nursing, pharmacy and applied medical science, to reflect a range of disciplinary perspectives while remaining relevant to the health sciences context. Recruitment is conducted by distributing an invitation through institutional email lists or via health sciences program coordinators, which provides a concise description of the study, outlines eligibility criteria, and includes the researcher’s contact information. Interested students undergo a screening process to verify that they meet the selection criteria and are willing to participate in a virtual interview.

**Table 1:** Participant demographics.

Participant ID	Gender	Program
P1	Female	Dentistry
P2	Male	Pharmacy
P3	Male	Applied Medical Sciences
P4	Female	Nursing

Ethical considerations are prioritized throughout the study, with informed consent obtained from all participants before the interviews. Participants are fully informed about the study’s purpose, their right to withdraw at any time without consequence, and the measures in place to protect their confidentiality, such as the use of pseudonyms and secure data storage. The study adheres strictly to institutional ethical guidelines, and approval is sought from the relevant ethics review board, ensuring that all procedures align with established standards for ethical research.

### Data collection

The study utilizes virtual semi-structured interviews conducted. An interview guide is carefully crafted, featuring open-ended questions designed to elicit detailed and reflective responses from participants. Examples of these questions include: “Can you describe your experiences with academic advising or support services at your institution?” “What aspects of the advice or support have been most helpful or unhelpful to you?” “How do factors like your program, campus, or personal background influence your experience with these services?” and “What improvements would you suggest for academic support services?” The guide is structured to allow flexibility, enabling the researcher to ask follow-up questions that probe deeper into participants’ responses and uncover richer insights. Interviews are scheduled at times convenient for participants, with a reminder sent 24 h in advance to ensure participation. At the beginning of each interview, the researcher reviews the consent form, explains the audio-recording process, and creates a comfortable environment to foster open dialogue. With participants’ permission, interviews are audio-recorded and transcribed verbatim to prepare for analysis. To maintain confidentiality, audio recordings and transcripts are stored on a password-protected device, accessible only to the research team, and transcripts are anonymized by replacing any identifiable information with pseudonyms.

### Data analysis

The study employs thematic analysis, as described by Braun and Clarke (36), to identify patterns and themes within the interview data, using an iterative process that encompasses familiarization, coding, theme development, and interpretation. Initially, the researcher engages in familiarization by thoroughly reading and re-reading the interview transcripts to gain a deep understanding of the data. Next, during the coding phase, meaningful segments of text are identified and labeled with initial codes that capture aspects of students’ experiences, perceptions, and demographic influences, such as “positive advisor interaction,” “language barriers,” or “campus-specific resources.” These codes are then organized into potential themes, such as “accessibility of support,” “personalized guidance,” or “cultural influences,” which are carefully reviewed and refined to ensure they accurately represent the data. Finally, in the interpretation stage, the identified themes are analyzed in relation to the study’s research questions, with connections made to the broader literature on student satisfaction and health sciences education to contextualize the findings.

## Results

Thematic analysis of the interview data revealed four major themes that capture the lived experiences of health sciences students regarding academic advice and support services: Accessibility and Availability of Support, Personalized Guidance and Advisor Engagement, Cultural and Language Influences, and Recommendations for Service Improvement.

### Accessibility and availability of support

Participants expressed varied experiences regarding the ease of accessing academic support services. Some students, particularly those based on the main campus, reported that support services were readily available and well-advertised through official channels. However, students from satellite campuses noted difficulties in accessing comparable resources, citing limited advisor availability and lack of awareness campaigns.

One nursing student from a satellite campus shared, *“Sometimes I feel like we are forgotten. The main campus has more services, but here, it’s hard to even know where to go for advice.”*

Additionally, participants highlighted the challenge of scheduling meetings with advisors due to their busy schedules. Pharmacy students, in particular, noted that their programs were highly demanding, and finding time to meet with advisors during working hours was difficult.

### Personalized guidance and advisor engagement

A recurring theme across interviews was the importance of receiving advice tailored to individual academic and career goals. Students who experienced personalized guidance described their interactions as highly beneficial, noting that advisors who took the time to understand their unique circumstances provided more practical and motivating advice.

A participant from the applied medical sciences program reflected, *“The best advice I received was from an advisor who really listened to my struggles and recommended courses that fit my interests and career plans. It made a big difference.”*

Conversely, students also shared negative experiences where advising sessions felt rushed and generic. Several participants perceived that some advisors followed a “one-size-fits-all” approach, offering standard advice without considering students’ personal goals or challenges.

### Cultural and language influences

Participants highlighted cultural expectations and language barriers as factors shaping their experiences with academic advising. Some female students indicated discomfort in discussing academic concerns openly with male advisors, reflecting cultural norms around gender interactions. This led to a preference for female advisors when discussing personal academic struggles.

Language proficiency also emerged as a significant influence, especially among students who struggled with English—the primary language used in academic advising sessions. A pharmacy student stated, *“Sometimes it’s hard to fully express myself in English when discussing complex issues. I feel like I cannot explain everything clearly, and this makes the advice less helpful.”*

These challenges often resulted in students feeling hesitant to seek further support, particularly when prior interactions had left them feeling misunderstood.

### Recommendations for service improvement

All participants offered suggestions for enhancing academic support services. The most frequently mentioned recommendation was the need for more structured and accessible workshops that introduce students to available resources early in their academic journey.

Additionally, students expressed interest in having more flexible meeting options, such as virtual advising sessions, to accommodate their busy academic schedules. Several participants also emphasized the value of mentorship programs where senior students could guide juniors through academic challenges.

One participant recommended, *“It would be really helpful to have regular workshops or even online modules that teach us how to plan our studies, manage stress, and make better academic decisions.”*

Finally, students stressed the importance of culturally sensitive advising practices, suggesting that advisors receive training to better understand and navigate cultural and language differences.

Prior research confirms that service quality dimensions strongly shape overall student satisfaction in higher education contexts (16, 22, 33).

## Discussion

This study explored the lived experiences of health sciences students regarding academic advice and support services, revealing several critical factors that influence their satisfaction and engagement. The findings offer valuable insights into how institutional resources, advisor practices, cultural dynamics, and language preferences shape the effectiveness of academic advising in health sciences education.

### Accessibility and institutional equity

The theme of accessibility highlights an important issue of institutional equity. While students on main campuses reported satisfactory access to support services, those from satellite campuses experienced significant challenges. This finding aligns with prior research indicating that disparities in resource allocation across campuses can negatively impact student engagement and satisfaction (7). Versfeld and Mapaling (8) further emphasize that academic advising practices in Global South contexts often face institutional and infrastructural constraints, limiting the availability of qualified advisors and accessible services. This reality underscores the importance of addressing systemic barriers and expanding support mechanisms to ensure equitable access for all students. Similar patterns have been observed across diverse contexts including Qatar, Saudi Arabia, Bhutan, and the UK, highlighting both regional variation and shared drivers of satisfaction (17, 18, 20, 28).

In response to these challenges, the role of academic advising—whether online, dual-credit, or professional programs—has consistently emerged as a critical factor influencing student engagement and perceived institutional support (25, 27, 29). several studies suggest that integrating technology into advising services may help bridge gaps in accessibility. Bilquise et al. (9) and Assayed et al. (10) highlight the growing acceptance of academic advising chatbots and conversational AI in higher education institutions, which can offer immediate, on-demand support, particularly for students in remote or underserved campuses. However, while these digital tools offer convenience, they should complement rather than replace human interaction, ensuring that students continue to receive personalized and empathetic guidance (11).

### The critical role of personalized guidance

The importance of personalized academic advising was consistently emphasized by participants. Students valued advisors who took the time to understand their individual academic goals and personal circumstances, which enhanced their motivation and academic planning. This finding supports the work of Tinto (2) and Afzal et al. (1), who argue that effective advising and mentoring play a crucial role in promoting student success, retention, and a sense of academic purpose.

Conversely, generic and transactional advising was perceived as ineffective, often leaving students feeling unsupported. Institutions must prioritize training advisors to adopt a more student-centered approach, focusing on active listening, empathy, and individualized planning strategies. Additionally, emerging technologies such as natural language processing can be leveraged to analyze student feedback and continuously improve the quality of advising services (12), ensuring that student voices inform the development of more responsive and relevant advising programs.

### Cultural sensitivity, language preferences, and EMI challenges

Language policy and English-medium instruction further complicate satisfaction outcomes, as cultural and linguistic alignment has been shown to affect both learning experience and institutional trust (6, 24, 30). While previous studies have acknowledged that students in EMI settings often prefer using their native language in certain contexts, the findings of this study suggest a deeper psychological and cognitive rationale for this behavior. Participants’ strong preference for Arabic during advising sessions should not be viewed merely as a cultural habit or linguistic convenience, but rather as a strategic response to the cognitive load, anxiety, and emotional vulnerability that are often heightened in English-only academic environments.

Communicating complex thoughts—such as academic confusion, emotional struggles, or long-term career concerns—in a second language requires significant mental effort. Research in second language acquisition consistently shows that using a non-native language under stress increases cognitive demand, reduces processing efficiency, and can trigger emotional inhibition (37). In such situations, reverting to one’s first language becomes a way to preserve clarity, control, and emotional safety.

In this study, several participants described difficulty articulating their academic concerns in English, even after years of language exposure. Their choice to use Arabic during advising was not just a linguistic preference but a way to regain expressive power and reduce the fear of being misunderstood or judged. This aligns with Sah and Karki (13), who noted that students in EMI programs often struggle with epistemic confidence when engaging in high-stakes conversations in a second language.

Moreover, from a psychological safety perspective, students appeared more willing to disclose academic doubts, emotional stress, or interpersonal concerns when the advising environment allowed for Arabic communication. This mirrors findings in the psychological safety literature (38), which emphasize that environments allowing for authenticity and reduced self-monitoring improve trust, participation, and help-seeking behavior.

Therefore, students’ linguistic choices can be interpreted as adaptive strategies to navigate an institutional structure that privileges English but does not always accommodate emotional or cognitive vulnerability. Their use of Arabic is not resistance—it is resilience.

Institutions implementing EMI policies should take this into account when designing academic support systems. Providing optional bilingual advising services or allowing code-switching in advising conversations may significantly reduce student anxiety, improve clarity in communication, and ultimately enhance the effectiveness of advising relationships. Doing so would move beyond linguistic inclusivity and toward cognitive empathy—acknowledging the real psychological cost of learning and help-seeking in a second language.

### Toward a strategic blended advising model: human empathy meets AI scalability

As higher education institutions embrace digital transformation, the promise of AI-driven tools—such as academic advising chatbots and virtual scheduling assistants—has become increasingly attractive. These tools offer round-the-clock availability, standardization, and cost efficiency, particularly in large or decentralized universities. However, this study’s findings reinforce a crucial boundary: while technology can extend access, it cannot replace the relational depth, contextual judgment, and emotional attunement that human advisors provide.

Rather than viewing AI as a full substitute, a more strategic approach lies in adopting a *blended advising model* grounded in task delegation. Under such a model, routine, transactional tasks—such as course registration, deadline reminders, or policy FAQs—are handled by conversational AI platforms. These tasks are rule-based, require minimal emotional nuance, and benefit from speed and scale. Delegating them to AI frees up valuable human advisor capacity to focus on higher-order responsibilities that students repeatedly identified as most meaningful: personalized academic guidance, empathetic listening, and culturally sensitive problem-solving.

This model is not simply about efficiency—it reflects a shift in priorities. Students in this study emphasized the importance of being heard, understood, and guided through complex decision-making processes. These are tasks that demand emotional intelligence, narrative understanding, and trust—qualities that current AI systems, despite advances, cannot fully replicate. Therefore, preserving the human dimension in advising is not optional; it is essential for addressing the emotional and identity-related challenges students face, especially in high-pressure health sciences programs.

Furthermore, AI-generated insights—such as predictive alerts based on course performance or disengagement patterns—can be used to proactively inform human advisors. In this way, technology supports—not replaces—human empathy by equipping advisors with timely, data-driven signals to intervene meaningfully.

Ultimately, the goal of blended advising should not be automation for its own sake, but intentional design: letting machines handle scale so that humans can offer care. A well-executed hybrid model can increase efficiency without compromising what students value most—empathetic, personalized, and culturally attuned guidance.

### Recommendations for enhancing support services

Participants offered practical suggestions for improving academic support, including the expansion of mentorship programs, the integration of virtual advising platforms, and the provision of structured workshops focusing on study planning, stress management, and academic success strategies. These recommendations are consistent with existing research advocating for holistic advising models that address not only academic concerns but also students’ emotional and psychological well-being (14, 15).

Implementing flexible and accessible advising formats, such as virtual consultations and online support modules, may help address scheduling conflicts and improve service reach, particularly for students in demanding programs like pharmacy and applied medical sciences. Additionally, introducing peer mentorship opportunities could foster a supportive academic community, helping students navigate common challenges through shared experiences.

### Limitations

While this exploratory study offers meaningful insights into the academic advising experiences of health sciences students, it is important to recognize several critical limitations. Foremost, the small sample size of four participants significantly constrains the generalizability of the findings. Due to the limited number of interviews, this study does not claim to have achieved thematic saturation. Instead, the results should be interpreted as preliminary, context-bound observations that offer early insights rather than representative conclusions.

The qualitative, case-based design was intentionally selected to explore individual perspectives in depth; however, the limited scale inherently increases the risk that the findings may reflect anecdotal experiences rather than broader patterns. As such, this study is best understood as a pilot investigation aimed at generating hypotheses and identifying key themes for further research.

Additionally, the study focused exclusively on student perspectives without incorporating the voices of academic advisors, support staff, or administrators. Including these additional viewpoints in future research would provide a more comprehensive understanding of the structural and relational dynamics that shape advising experiences.

Lastly, the research was conducted within a single national and institutional context, which may limit the transferability of the findings to other English-medium institutions with different cultural or structural characteristics.

### Conclusion and implications

This study provided valuable insights into the lived experiences of health sciences students regarding academic advising and support services within an English as a Medium of Instruction (EMI) context. The findings highlight the critical importance of accessibility, personalized guidance, cultural sensitivity, and language considerations in shaping students’ satisfaction with academic support. While technological solutions such as AI-powered advising platforms are becoming increasingly prominent, this study reinforces the continued need for human-centered, empathetic advising practices that address students’ complex academic, emotional, and cultural needs.

Other studies underline the importance of psychological well-being, sense of belonging, and willingness to seek help, particularly in online learning settings (21, 26, 34, 35). By acknowledging the significant role of language preferences and cultural dynamics, institutions can create more inclusive advising environments that foster student engagement, well-being, and academic success. Additionally, adopting a blended advising model—combining the efficiency of digital tools with the depth of human interaction—can help institutions meet the evolving needs of diverse student populations, particularly in health sciences programs where academic demands are high and career planning is complex.

### Implications for practice and future research

The findings of this study have important implications for both academic practice and future research in the field of higher education, particularly within EMI environments. First, there is a clear need for institutions to enhance the cultural and linguistic responsiveness of their academic advising services. Given that many students expressed a strong preference for using their native Arabic language when discussing complex academic and personal issues, universities should invest in training programs that help advisors develop greater cultural sensitivity and awareness of language barriers. This includes equipping advisors with the skills necessary to navigate cross-cultural communication effectively and ensuring that advising services offer flexible language options where possible. Such initiatives can foster a more inclusive and supportive advising environment, ultimately improving students’ academic engagement and satisfaction.

In addition, higher education institutions should move toward implementing blended advising models that integrate both technological solutions and human-centered approaches. While AI-driven tools such as chatbots and virtual advising platforms offer increased accessibility and efficiency—especially for answering routine queries—this study reinforces that human interaction remains critical for addressing students’ deeper academic concerns and emotional well-being. Blended models can help institutions meet the evolving expectations of modern students by providing immediate, accessible support through digital platforms while preserving the relational depth and empathy that only face-to-face or personalized virtual advising can offer.

Another important implication is the need to expand access to virtual advising services, particularly for students in health sciences programs who face demanding academic schedules and for those located on satellite campuses with limited access to on-site advisors. Providing flexible virtual advising options, including scheduled online consultations and interactive workshops, can help bridge the gap in service accessibility and ensure that all students receive timely and relevant academic support regardless of their physical location.

Furthermore, institutions should consider developing structured peer mentorship programs that leverage the experiences of senior students to guide and support their junior peers. Such programs not only provide practical academic advice but also contribute to building a stronger sense of community and belonging among students, which has been shown to positively impact retention and academic performance.

Finally, future research should build on these findings by exploring academic advising from a more holistic perspective, incorporating the voices of both students and academic advisors. Larger, more diverse samples would help to validate these results and offer a broader understanding of how cultural, linguistic, and institutional factors interact to shape advising experiences. Longitudinal studies examining the effectiveness of blended advising models and culturally responsive practices over time would also provide valuable insights for policy development and the continuous improvement of academic support services.

## Data Availability

The raw data supporting the conclusions of this article will be made available by the authors, without undue reservation.
